# Volatile-mediated plant–plant interactions: volatile organic compounds as modulators of receiver plant defence, growth, and reproduction

**DOI:** 10.1093/jxb/erab487

**Published:** 2021-11-17

**Authors:** Agnès Brosset, James D Blande

**Affiliations:** Department of Environmental and Biological Sciences, University of Eastern Finland, Yliopistonranta 1 E, P.O. Box 1627, Kuopio FIN-70211, Finland; Department of Environmental and Biological Sciences, University of Eastern Finland, Yliopistonranta 1 E, P.O. Box 1627, Kuopio FIN-70211, Finland; Helmholtz Zentrum München, Germany

**Keywords:** Defence, green leaf volatiles, growth, photosynthesis, plant–plant communication, primary metabolism, priming, reproduction, secondary metabolism, terpenes, volatile organic compounds

## Abstract

It is firmly established that plants respond to biotic and abiotic stimuli by emitting volatile organic compounds (VOCs). These VOCs provide information on the physiological status of the emitter plant and are available for detection by the whole community. In the context of plant–plant interactions, research has focused mostly on the defence-related responses of receiver plants. However, responses may span hormone signalling and both primary and secondary metabolism, and ultimately affect plant fitness. Here we present a synthesis of plant–plant interactions, focusing on the effects of VOC exposure on receiver plants. An overview of the important chemical cues, the uptake and conversion of VOCs, and the adsorption of VOCs to plant surfaces is presented. This is followed by a review of the substantial VOC-induced changes to receiver plants affecting both primary and secondary metabolism and influencing plant growth and reproduction. Further research should consider whole-plant responses for the effective evaluation of the mechanisms and fitness consequences of exposure of the receiver plant to VOCs.

## Teaser

This review highlights the importance of volatile organic compounds (VOCs) in plant–plant interactions, covering the defence, growth, and reproduction responses in recipient plants upon VOC exposure.

## Introduction

Plants produce and emit a diverse array of volatile organic compounds (VOCs) as defences against biotic and abiotic stresses and to interact with their environment (Dudareva *et al.*, 2013). Upon damage by herbivores, plants emit *de novo*-produced VOCs to defend against the attacker; these VOCs can also act as a rapid warning signal to prime defences in undamaged parts of the same plant and undamaged neighbouring plants (McCall *et al.*, 1994; Engelberth *et al.*, 2004; Heil and Silva Bueno, 2007). This phenomenon, referred to as a plant–plant interaction or plant–plant communication, was first documented in the early 1980s in studies that were the subject of extensive debate (Baldwin and Schultz, 1983; Rhoades, 1983). The scientific discourse that ensued threw doubt on the conclusions (Fowler and Lawton, 1985) and temporarily slowed progress in the field (Karban *et al.*, 2014*a*). However, a wealth of studies since has provided a solid literature base supporting the paradigm that plants release VOCs that can be detected by and elicit responses in their neighbours (Karban *et al.*, 2014*b*).

A common observation has been that exposure to herbivore-induced plant volatiles (HIPVs) elicits changes in a receiver plant that decreases the cumulative seasonal herbivore damage to that receiver (Dolch and Tscharntke, 2000; Tscharntke *et al.*, 2001; Karban and Maron, 2002; Karban *et al.*, 2006). The frequency with which this observation has been reported provides compelling evidence that volatile-mediated plant–plant interactions are ecologically significant (Karban *et al.*, 2014*b*), and great progress has been made in elucidating the integration of VOCs into plant defence mechanisms (Erb, 2019; Ye *et al.*, 2019). These mechanisms encompass the detection of VOCs and the triggering of sophisticated molecular pathways (Ye *et al.*, 2019), and the associational resistance facilitated through chemical camouflage (Himanen *et al.*, 2010; Mofikoya *et al.*, 2017; Bui *et al.*, 2021). There is now less debate about the phenomenon of volatile-mediated plant–plant interactions, but there remain a lot of questions about how sophisticated, flexible, or specific the interactions are.

Many studies of biotic-stress-induced plant–plant interactions have focused on defence-related responses of receiver plants—the common assumption being that plants can pre-empt a potential herbivore attack and ready or initiate their defences in preparation (e.g. Heil and Kost, 2006; Kost and Heil, 2006; Frost *et al.*, 2007). The rationale behind this strategy is logical if we consider that volatile-mediated plant–plant interactions could have evolved as an artifact of volatile-mediated within-plant defence coordination via volatiles (Heil and Silva Bueno, 2007; Frost *et al.*, 2007; Heil and Karban, 2010; Li and Blande, 2017). Within-plant signalling via volatiles could have evolved to overcome vascular constraints, which may be extensive in highly branched modular plants (Frost *et al.*, 2007). However, responses may be broader than being defence related, and may span receiver plant hormone signalling and primary and secondary metabolism, and ultimately affect plant fitness. To gain a complete picture of biotic-stress-induced plant–plant interactions, we need to expand research beyond the common focus on plant defence to encompass the effects of VOC exposure on non-defence-related parameters.

In this review, we present an overview of plant–plant interactions with an emphasis on receiver plants. The review begins with a brief description of recent advances in the perception, uptake, and conversion of VOCs and then addresses recent research on passive volatile-mediated plant–plant interactions. The focus then develops to cover defence-related changes induced or primed by VOCs in receiver plants, before synthesizing literature that addresses how VOCs influence non-defence-related parameters. We found that few studies have addressed the effect of VOCs on non-defence-related parameters, with those studies suggesting that VOCs might affect gas exchange and nutrient assimilation, influencing the resources allocated to primary and secondary metabolism, and ultimately affecting plant growth, reproduction, and defence. We finish with a consideration of the ecological consequences of responding to VOCs and conclude with a summary and suggestions for future research. Our aim is to emphasize the need to consider whole-plant responses for effective evaluation of the mechanisms and consequences of volatile-mediated plant–plant interactions across mechanistic and ecological levels of organization.

## Perception, uptake, and conversion of VOCs

It has typically been concluded in the literature that the mechanisms of detection of volatiles by plants are poorly understood (e.g. Hemachandran *et al.*, 2017); however, there has been a lot of progress in this area and knowledge of at least partial mechanisms has been accumulating. Information on the identities of molecules eliciting responses in receiver plants has steadily grown (see Table 1), while the early stages of responses have become increasingly clear (Erb, 2019). One of the reasons that mechanisms have been viewed as elusive is the general lack of VOC receptors that have been identified in plants. Nevertheless, at least 38 different VOCs have been implicated as providing a form of between-plant cue (Table 1). This suggests that there are likely to be mechanisms other than through specific molecule receptors that initiate the process of volatile detection by plants. It has been proposed that VOCs could be considered as damage-associated molecular patterns, which are tissue-derived signals of damage that initiate cellular signalling cascades (reviewed by Quintana-Rodriguez *et al.*, 2018; Meents and Mithöfer, 2020). In this capacity, VOCs may trigger plant immunity and have roles in protection against herbivores and disease.

**Table 1.** Known VOCs inducing direct responses in plants and/or priming of defences upon biotic or abiotic stress

Compound	Species	Exposure time	Induced responses in the receiver plants	Primed responses upon stress in the receiver plants	Refs
*Aldehydes*					
Acrolein	*Arabidopsis thaliana*	3 h	Increases the expression of defensive genes similar to those expressed upon *Pseudomonas syringae* infection and induces the production of jasmonates		Alméras *et al.*, 2003
Methacrolein	*Nicotiana attenuata*	72 h		Enhances the production of trypsin proteinase inhibitors in response to subsequent herbivory by *Manduca sexta* caterpillars	Kessler *et al.*, 2006
Nonanal	*Phaseolus lunatus*	6h and 24 h		All concentrations and times tested reduce infection by the bacterial pathogen *P. syringae*	Girón-Calva *et al.*, 2012
*Benzenoids*					
Benzothiadiazole	*P. lunatus*	Spraying		Increases expression of *PR-2* gene and increases resistance to *P. syringae*	Yi *et al.*, 2009
Indole	*Camellia sinensis*	48 h	Directly induces salicylic acid (SA) production	Increases resistance to *Ectropis obliqua* larvae. Up-regulates genes involved in Ca^2+^ signalling and mitogen-activated protein kinase and increases the production of phytohormones after herbivory	Ye *et al.*, 2021
	*Oryza sativa*	12 h	Increases the accumulation of 12-oxophytodienoic acid and up-regulates LRR-RLKs gene expression	Increases the expression of early defence signalling genes and enhances jasmonic acid (JA) production upon herbivory. Decreases *Spodoptera frugiperda* larval growth and damage	Ye *et al.*, 2019
	*Zea mays*	12 h	Increases abscisic acid (ABA) production	Increases ABA and jasmonic acid isoleucine production upon wounding and application of *Spodoptera littoralis* regurgitant	Erb *et al.*, 2015
	*Z. mays*	16 h		Increases jasmonate production and volatile abundance, and induces defence gene expression after herbivory	Hu *et al.*, 2019
(+)-Menthofuran	*Cucumis sativus*	Perfusion	Induces *V*_m_ depolarization		Maffei *et al.*, 2001
Methyl salicylate (MeSA)	*P. lunatus*	6h and 24 h		Reduces *P. syringae* infection	Girón-Calva *et al.*, 2012
*Green leaf volatiles*					
(*E*)-2-Hexenal	*A. thaliana*	15–20 min	Rapidly promotes cytosolic calcium ([Ca^2+^]cyt) transients and induces superoxide production		Asai *et al.*, 2009
	*A. thaliana*	24 h	Up-regulates defence-related genes	Increases plant resistance to the necrotrophic fungal pathogen *Botrytis cinerea*	Kishimoto *et al.*, 2005
	*Lolium temulentum*	1–60 min	Activates mitogen-activated protein kinases		Dombrowski *et al.*, 2018
	*N. attenuata*	72 h		Enhances the production of trypsin proteinase inhibitors to subsequent *M. sexta* caterpillar feeding	Kessler *et al.*, 2006
	*Solanum lycopersicum*	Perfusion	Induces *V*_m_ depolarization and increases [Ca^2+^]cyt		Zebelo *et al.*, 2012
(*Z*)-3-Hexenal	*A. thaliana*	24 h	Up-regulates defence-related genes	Increases plant resistance to the necrotrophic fungal pathogen *B. cinerea*	Kishimoto *et al.*, 2005
	*S. lycopersicum*	Perfusion	Induces *V*_m_ depolarization and increases [Ca^2+^]cyt		Zebelo *et al.*, 2012
	*Z. mays*	30 min	Increases JA production and quantities of VOCs emitted	Increases JA production and quantities of VOCs emitted after *Spodoptera exigua* regurgitant application	Engelberth *et al.*, 2004
	*Z. mays*	3–48 h	Up-regulates defensive genes and increases MeSA emissions		Farag *et al.*, 2005
(*E*)-2-Hexenol	*A. thaliana*	15–20 min	Promotes [Ca^2+^]cyt transients		Asai *et al.*, 2009
	*L. temulentum*	1–60 min	Activates mitogen-activated protein kinases		Dombrowski *et al.*, 2018
(*Z*)-3-Hexenol(*E*)-3-Hexenol	*L. temulentum*	1–60 min	Activates mitogen-activated protein kinases		Dombrowski *et al.*, 2018
	*Z. mays*	30 min	Increases JA production and quantities of VOCs emitted	Increases JA production and quantities of VOCs emitted after *S. exigua* regurgitant application	Engelberth *et al.*, 2004
	*Z. mays*	20–60 min	Up-regulates genes involved in transcriptional regulation and Ca^2+^ and lipid signalling		Engelberth *et al.*, 2013
	*Z. mays*	14 h	Increases the quantity of VOCs and induces emissions of VOCs associated with herbivory, such as (*Z*)-3-hexenyl acetate, (3*E*)-DMNT, and sesquiterpenes		Ruther and Kleier, 2005
(*Z*)-3-Hexenyl acetate(*E*)-3-Hexenyl acetate	*P. lunatus*	24 h	Increases the production of extrafloral nectar		Heil *et al.*, 2008
	*Populus deltoides* × *nigra*	16 h		Induces higher concentrations of JA and linolenic acid upon feeding by *Lymantria dispar* larvae. Up-regulates the expression of direct defence-related genes, and induces stronger and quicker emissions of terpenes upon larvae feeding	Frost *et al.*, 2008
	*S. lycopersicum*	Perfusion	Induces *V*_m_ depolarization and increases [Ca^2+^]cyt		Zebelo *et al.*, 2012
	*Triticum aestivum*	16 h		Increases H_2_O_2_ production and increases activity levels of ROS-scavenging enzymes. Lowers infection rate by the fungus *Fusarium graminearum* and necrotic lesions induced by the fungus	Ameye *et al.*, 2015; Van Meulebroek *et al.*, 2020
	*Z. mays*	30 min	Increases JA production and the quantities of VOCs emitted	Increases JA production and quantities of VOCs emitted after *S. exigua* regurgitant application	Engelberth *et al.*, 2004
	*Z. mays*	1.5–4 h	Increases the expression of cold-stress-related genes	Reduces damage after cold stress	Cofer *et al.*, 2018
	*Z. mays*	24 h	Directly induces defence gene expression in the absence of herbivory	Increases jasmonate production and volatile abundance, and induces defence gene expression after herbivory	Hu *et al.*, 2019
(*E*)-2-Hexenyl acetate5-Hexenyl acetate(*Z*)-3-Hexenyl isovalerate(*Z*)-3-Hexenyl butyrate	*P. lunatus*	24 h	Increases the production of extrafloral nectar		Heil *et al.*, 2008
*Jasmonates*					
Methyl jasmonate (MeJA)	*Gossypium hirsutum*	16 h	Increase emissions of (*Z*)-3-hexenyl acetate, (*E*)-β-ocimene, linalool, (3*E*)-DMNT, (*E*,*E*)-α-farnesene, (*E*)-β-farnesene, and (*E*,*E*)-TMNT		Rodriguez-Saona *et al.*, 2001
	*S. lycopersicum*	24 h	Induces proteinase inhibitor I and II proteins		Farmer and Ryan, 1990
	*Z. mays*	3–24 h	Increases the expression of defensive genes		Farag *et al.*, 2005
*Ketones*					
(*Z*)-Jasmone	*Z. mays*	24 h	Reduces susceptibility to the leafhopper *Cicadulina storeyi*	Increases emission of the sesquiterpenes (*E*)-(1R,9S)-caryophyllene, (*E*)-α-bergamotene, (*E*)-β-farnesene, and (*E*)-DMNT upon insect feeding	Oluwafemi *et al.*, 2013
(–)-Menthone	*C. sativus*	Perfusion	Induces *V*_m_ depolarization		Maffei *et al.*, 2001
Methyl vinyl ketone	*A. thaliana*	3 h		Increases the expression of defensive genes upon *P. syringae* infection	Alméras *et al.*, 2003
(+)-Pulegone	*C. sativus*	Perfusion	Induces *V*_m_ depolarization		Maffei *et al.*, 2001
Vinyl ketone	*A. thaliana*	3 h		Increases the expression of defensive genes upon *P. syringae* infection	Alméras *et al.*, 2003
*Terpenes*					
Isoprene	*A. thaliana*	72 h		Functions through SA signalling to prime plant defence and reduce the growth of the biotrophic pathogen *P. syringae*	Frank *et al.*, 2021
α-Pinene	*A. thaliana*	2 h	Increases pinII-promoter activity		Godard *et al.*, 2008
	*A. thaliana*	15–20 min	Promotes [Ca^2+^]cyt transients		Asai *et al.*, 2009
	*A. thaliana*	72 h	Induces the accumulation of reactive oxygen species (ROS), and the expression of salicylic acid (SA)-and systemic acquired resistance (SAR)-associated genes		Riedlmeier *et al.*, 2017
	*S. lycopersicum*	Perfusion	Induces *V*_m_ depolarization		Zebelo *et al.*, 2012
β-Pinene	*A. thaliana*	72 h	Induces the accumulation of ROS, and the expression of SA-and SAR-associated genes		Riedlmeier *et al.*, 2017
	*A. thaliana*	15–20 min	Promotes [Ca^2+^]cyt transients		Asai *et al.*, 2009
Cumene	*Brassica nigra*	5 d		Reduces larval biomass	Pashalidou *et al.*, 2020
Myrcene	*A. thaliana*	2 h	Increases pinII-promoter activity and up-regulates genes associated with response to biotic or abiotic stress, defence, and transcription factors		Godard *et al.*, 2008
	*A. thaliana*	15–20 min	Promotes [Ca^2+^]cyt transients		Asai *et al.*, 2009
Limonene	*A. thaliana*	2 h	Increases pinII-promoter activity		Godard *et al.*, 2008
1,8-Cineole	*A. thaliana*	2 h	Increases pinII-promoter activity		Godard *et al.*, 2008
	*Artemisia tridentata*	24 h		Reduces herbivore damage	Shiojiri *et al.*, 2015
Linalool	*A. thaliana*	2 h	Increases pinII-promoter activity		Godard *et al.*, 2008
	*S. lycopersicum*	24h		Plants from the variety Moneymaker increased JA levels and expression of the *PI-I* gene 6 and 12 h after *S. exigua* caterpillar regurgitant treatment	Zhang *et al.*, 2020
(*E*)-β-Ocimene	*A. thaliana*	15–20 min	Promotes [Ca^2+^]cyt transients		Asai *et al.*, 2009
	*A. thaliana*	2 h	Increases pinII-promoter activity and up-regulates genes associated with response to biotic or abiotic stress, defence, and transcription factors		Godard *et al.*, 2008
	*Brassica pekinensis*	24 h		Increases the concentration of glucosinolates in leaves upon *Myzus persicae* infection and reduces the performance of *M. persicae*	Kang *et al.*, 2018
	*P. lunatus*	3–72 h	Up-regulates defence-related genes	Induces greater emissions of VOCs such as MeSA and (*E*)-DMNT after 1 d of feeding by *Tetranychus urticae*. Increases plant attraction to the predatory mite *Phytoseiulus persimilis*	Arimura *et al.*, 2000*a*; Arimura *et al.*, 2012, Muroi *et al.*, 2011
	*S. lycopersicum*	24 h		Plants from the variety Moneymaker exposed to linalool showed increased expression of the *PI-I* and *PI-II* genes at 12 h after *S. exigua* caterpillar regurgitant treatment	Zhang *et al.*, 2020
	*S. lycopersicum*	72 h	Induces emissions of VOCs. Decreases susceptibility to *Macrosiphum euphorbiae* aphids and increases attractivity to *Aphidius ervi* parasitoids		Cascone *et al.*, 2015
	*Z. mays*	72 h		Induces greater emissions of VOCs after 1 d of feeding by *Mythimna separata* larvae and reduces larval weight gain	Muroi *et al.*, 2011
*allo*-Ocimene	*A. thaliana*	24 h	Up-regulates defence-related genes	Increases plant resistance to the necrotrophic fungal pathogen *B. cinerea*	Kishimoto et al., 2005, 2006
(*E*)-DMNT	*A. thaliana*	15–20 min	Promotes [Ca^2+^]cyt transients		Asai *et al.*, 2009
	*C. sinensis*	6 h		Decreases the amount of leaf eaten by *Ectropis obliqua*. Increases JA and SA contents and up-regulates defence-related genes	Jing *et al.*, 2021
	*Ipomoea batatas*	3 h		Increases trypsin inhibitory activity induced by *Spodoptera litura* feeding and reduces larval weight	Meents *et al.*, 2019
	*P. lunatus*	3h and 24 h	Increases the expression of defence-related genes		Arimura *et al.*, 2000*b*
(*E*)-TMTT	*P. lunatus*	24 h	Increases the expression of defence-related genes		Arimura *et al.*, 2000*b*
(–)-Menthol	*C. sativus*	Perfusion	Induces *V*_m_ depolarization and increases [Ca^2+^]cyt		Maffei and Camusso, 2001; Maffei *et al.*, 2001
(+)-Neomenthol	*C. sativus*	Perfusion	Induces *V*_m_ depolarization		Maffei *et al.*, 2001
(*E*)-Nerolidol	*C. sinensis*	0.5–2 h	Activates mitogen-activated protein kinase, induces H_2_O_2_ burst, and increases JA and SA contents. Reduces susceptibility to the pathogen *Colletotrichum fructicola*	Increases resistance to *Empoasca onukii*	Chen *et al.*, 2020
β-Caryophyllene	*S. lycopersicum*	Perfusion	Induces *V*_m_ depolarization		Zebelo *et al.*, 2012
	*A. tridentata*	24 h		Reduces herbivore damage	Shiojiri *et al.*, 2015
	*A. thaliana*	72 h		Functions through JA signalling to prime plant defence and reduce growth of the biotrophic pathogen *P. syringae*	Frank *et al.*, 2021

An active volatile-mediated interaction between plants requires the signalling VOCs to traverse the plant cuticle. The cuticle is the final barrier for VOCs to cross for release to the atmosphere (Liao *et al.*, 2021); it is, similarly, a barrier to the entry of VOCs into receiver plants (Noe *et al.*, 2008). The uptake of VOCs depends on their physicochemical properties and the properties of the plant surface. For example, limonene, a characteristic hydrophobic monoterpene that is readily incorporated into cell membranes, is taken up by plants, with the degree of uptake scaling positively with lipid content (Noe *et al.*, 2008).

A series of studies has shown that VOCs taken up by plants can be converted into new compounds, with alterations to compounds from several different groupings described so far. Green leaf volatiles (GLVs), which have been implicated as mediators of plant–plant interactions in several studies (see Table 1), were shown to be taken up by tomato plants (Sugimoto *et al.*, 2014). Interestingly, exposure to tomato volatiles induced by common cutworm feeding, a major component of which is (*Z*)-3-hexanol, resulted in receiver plants having higher concentrations of (*Z*)-3-vicianoside. Labelling (*Z*)-3-hexanol, an aglycone of (*Z*)-3-vicianoside, with deuterium enabled the passage of the compound in receiver plants to be followed and showed that glycosylation of the up-taken aglycone was the mechanism underpinning (*Z*)-3-vicianoside accumulation (Sugimoto *et al.*, 2014). A recent study of wheat plants demonstrated that another GLV, (*Z*)-3-hexenyl acetate, a compound frequently linked to plant–plant interactions, is also taken up and metabolized by plants (Ameye *et al.*, 2020). The authors used metabolomics to show that exposure to (*Z*)-3-hexenyl acetate resulted in an increase in oxidative stress, modulation of the phenylpropanoid pathway, and a subsequent induction of glycosylation processes. These observations provide strong indications that GLV uptake and conversion could be involved in the overall responses of receiver plants as a result of plant–plant interactions.

In addition, it has been observed that exposure to (*E*)-nerolidol, a volatile sesquiterpene alcohol and precursor to (3*E*)-4,8-dimethyl-1,3,7-nonatriene (DMNT), increases the DMNT emissions of *Achyranthes bidentata* receiver plants under conditions of herbivore feeding or exposure to methyl jasmonate (MeJA) (Tamogami *et al.*, 2011). DMNT is a common HIPV, which has been shown to be primed in volatile-mediated plant–plant interactions (Giron-Calva *et al*., 2014). Deuterium labelling showed that (*E*)-nerolidol taken up by receiver plants was converted into DMNT (Tamogami *et al.*, 2011). Interestingly, nerolidol uptake and conversion into nerolidol glucoside has been observed in tea plants (*Camellia sinensis*) (Zhao *et al.*, 2020). Nerolidol exposure also correlated with a decrease in malondialdehyde content and increased resistance to cold stress (Zhao *et al.*, 2020). These studies suggest an important role of uptake and conversion of VOCs on the defence properties of receiver plants to both abiotic and biotic stressors, with responses potentially underpinning the observations of defence priming in some species.

The plant hormone jasmonic acid (JA) and its methyl ester, MeJA, are important regulators of plant responses to stress. Airborne MeJA has been shown to be taken up by plants and converted into JA, jasmonoyl isoleucine, and jasmonoyl leucine (Tamogami *et al.*, 2008). Additional conversion products were described by Oki *et al.* (2019). These conversions initiate signal transduction leading to the emission of VOCs (Tamogami *et al.*, 2008). The role of jasmonates in plant stress responses, growth, and development have been extensively reviewed (e.g. Wasternack, 2007; Wang *et al.*, 2021) and, although integral to mechanisms of plant–plant interactions, will not be considered in detail here.

Interestingly, similar mechanisms of uptake and conversion of VOCs have also been observed in roots. A study of co-cultivated rye (*Secale cereale*) and hairy vetch (*Vicia villosa*) showed that benzoxazinoids released into the rhizosphere by rye are taken up by neighbouring vetch plants and translocated to shoots (Hazrati *et al.*, 2020). However, the full mechanism underlying this observation is currently lacking. Literature documenting the uptake of chemical compounds by above- and belowground plant parts and conversion into biologically active molecules highlights the entry of chemical compounds into plants as an important part of an active plant–plant interaction.

## Passive deposition of VOCs on receiver leaves and chemical camouflage

Volatile-mediated plant–plant interactions can occur via active and passive mechanisms (Li and Blande, 2015). By definition, an active interaction requires a response by the receiver plant, which can usually be observed as molecular or physiological changes (see below, Defence responses induced and primed by VOCs) (Arimura *et al.*, 2000*a*). Passive interactions involve the sequestration or adherence of volatiles to the surfaces of a receiver plant, with further changes not required (Himanen *et al.*, 2010; Camacho-Coronel *et al.*, 2020). Both mechanisms of interaction may result in a change in the volatile profiles released by receiver plants, as either an immediate response, an effect of changes in abiotic conditions, or a primed response to stress (Himanen *et al.*, 2015; Li, 2016; Mofikoja *et al*., 2018). Adsorption of VOCs to plant surfaces can occur between conspecific plants (Li and Blande, 2015) or in heterospecific associations (e.g. Himanen *et al.*, 2010). Where VOCs from a strongly emitting plant adsorb to the surfaces of weaker emitting plants and provide associational resistance to pests or disease, the term ‘chemical camouflage’ may be used (Bui *et al.*, 2021).

It is notable that several studies have shown important ecological roles for passively mediated interactions, which involve the adsorption of chemicals to surfaces of plants. The chemicals most frequently linked to passive interactions are the sesquiterpenes, which are often referred to as semi-volatile compounds (Mofikoya *et al.*, 2019). Passive interactions have been shown to occur in conspecific and heterospecific associations, which can lead to beneficial or detrimental effects on receiver plants. Herbivore-induced sesquiterpenes emitted by broccoli plants (*Brassica oleracea* var*. italica*) have been shown to render conspecific neighbours more susceptible to oviposition (Li and Blande, 2015), whereas sesquiterpenes and sesquiterpene alcohols emitted by *Rhododendron tomentosum* have been shown to stick to neighbouring birch and confer greater resistance to herbivores (Himanen *et al.*, 2010).

Glandular trichomes are significant reservoirs of sesquiterpenes, which are stored in high concentrations and prevented from entering the subcellular space, where they could be toxic (Tissier *et al.*, 2017). When the trichomes are broken, for example, by insect feeding, or when temperatures are high, large quantities of sesquiterpenes can be emitted (Mofikoya *et al.*, 2019). After release, these volatiles can reach a receiver plant and be deposited on the plant surface. When temperatures are adequate for their volatility, the newly acquired volatiles can be re-emitted by the receiver plant in addition to its own characteristic blend of volatiles (Mofikoya *et al.*, 2019). Leaf surface characteristics, air temperature, and leaf surface temperature, as well as the physico-chemical properties of volatiles, are important factors in determining the deposition and re-release of VOCs from leaf surfaces (Schaub *et al.*, 2010; Niinemets *et al.*, 2014).

The adherence and release of *R. tomentosum* volatiles by neighbouring mountain birch trees in the subarctic has an important temperature-related component (Himanen *et al.*, 2010; Mofikoya *et al.*, 2019). The re-release of volatiles could potentially have a camouflaging effect, making plants less attractive to foraging herbivores. However, the adsorption or sequestration of volatiles by surface waxes has been shown to have some direct effects on receiver plant defences. Using cuticular-wax-covered microscope slides, Li and Blande (2015) showed that sesquiterpenes adsorb to surfaces and affect the oviposition choices by *Plutella xylostella* moths, whereas Camacho-Coronel *et al.* (2020) showed that 20 different VOCs were sequestered by wax-covered slides, with 18 of them significantly reducing conidia germination of the fungal pathogen *Colletotrichum lindemuthianum*. These studies indicate that passive volatile-mediated interactions can have significant roles in structuring the interactions of receiver plants with other organisms in the community.

## Defence responses induced and primed by VOCs

### VOC-induced responses in receiver plants

#### Early response events

Early studies on volatile-mediated plant–plant interactions focused on induced responses, the changes occurring in receiver plants in response to a volatile cue. A number of defence-related responses were observed in above- and belowground plant parts, including both those related to direct defence against the invader (e.g. Arimura *et al.*, 2000*a*) and indirect defence through the attraction of beneficial insects (e.g. Kost and Heil, 2006) (Table 1).

The earliest detectable event in the plant response to VOC exposure is a change in the plasma membrane potential of exposed cells (Maffei *et al.*, 2004). The plasma membrane of cells is the only cellular compartment to have direct contact with the extracellular medium through which a volatile arrives at a receiver plant. Once the volatiles are recognized at the membrane a further cascade of events can be initiated, leading to adapted responses. VOCs have been shown in a series of electrophysiological studies to elicit changes in the transmembrane potential, either through hyperpolarization (increases) or depolarization (decreases), which correlate with the activation of signal transduction pathways that lead to changes in gene expression (Zebelo and Maffei, 2016). Membrane depolarization is a fast electrical signal (action potential) that travels through the entire plant from the point of origin of the perceived input (Maffei and Bossi, 2006). It can function in systemic responses, allowing quick but non-specific signals to propagate through the entire plant (Maffei *et al.*, 2007). Changes in plasma transmembrane potential (*V*_m_) result in a change in ion fluxes through the membrane (Maffei *et al.* 2004; Zebelo and Maffei, 2015), most notably the movement of calcium (Ca^2+^) into the cytosol. Zebelo *et al.* (2012) exposed tomato leaves to different products of the lipoxygenase pathway (commonly known as GLVs)—monoterpenes and sesquiterpenes—and found that all volatiles tested depolarized the *V*_m_. However, determination of Ca^2+^ influx showed that (*Z*)-3-hexenyl acetate and (*E*)-2-hexenal prompted a strong Ca^2+^ signature in treated leaves, whereas α-pinene and β-caryophyllene did not induce a Ca^2+^ flux (Zebelo *et al.*, 2012). The Ca^2+^ ion is a secondary messenger in numerous plant signalling pathways and is rigorously regulated across the plasma membrane by passive fluxes (Ca^2+^ channels) and active transport (Ca^2+^ transporters) (Lecourieux *et al.*, 2006). It is an important secondary messenger of plant immune responses, and import of Ca^2+^ into the cytosol acts as a signal that may induce cellular responses involving the production of hydrogen peroxide (H_2_O_2_) (Hepler, 2005). Exposure to several GLVs and terpenes was found to increase cytosol Ca^2+^ concentrations prior to herbivore feeding (Table 1). Hence, Ca^2+^ is an important indicator of a significant active plant–plant interaction.

Similar electrophysiological changes in the plasma cell membranes were found in plant roots. For example, several compounds, including (+)-menthofuran, (+)-pulegone, (+)-neomenthol, (–)-menthol, and (–)-menthone, were found to depolarize root cell membranes of cucumber seedlings (Maffei *et al.*, 2001). Of these compounds, (–)-menthol was found to increase the cytosolic Ca^2+^ concentration of the root cells (Maffei and Camusso, 2001). This observation suggests a similar detection mechanism in the root apices to that observed in the leaves, whereby high concentrations of VOCs triggered a depolarization of membranes.

In response to herbivore wounding, pathogen attack, or insect-derived elicitors, the production of superoxide (O_2_^–^) and H_2_O_2_ can act as a local signal for hypersensitive cell death and as a systemic signal inducing defensive genes in adjacent cells (Orozco-Cárdenas *et al.*, 2001; Maffei *et al.*, 2006; Smirnoff and Arnaud, 2019). Similar responses were found when *Arabidopsis thaliana* plants were exposed to α-pinene and β-pinene, which induced the accumulation of O_2_^–^ and up-regulated systemic acquired resistance (SAR)-mediated genes (Riedlmeier *et al.*, 2017).

#### Late response events

**Fig. 1.**
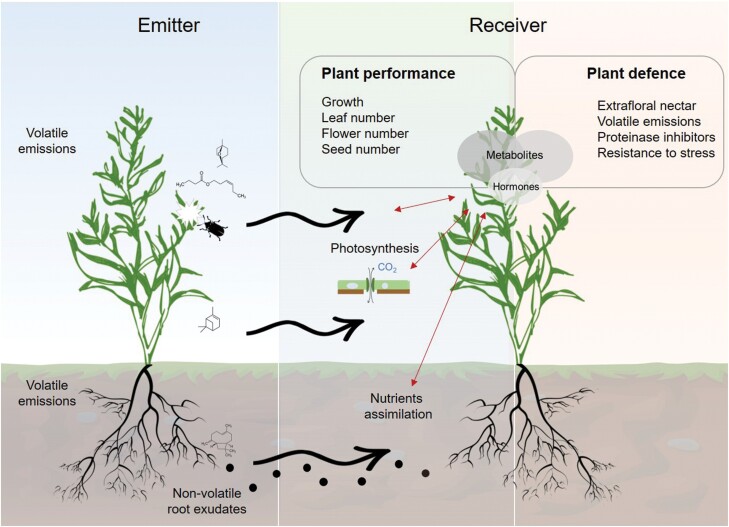
Summary of known changes occurring in receiver plants induced by volatile cues. By influencing the rate of photosynthesis, nutrient assimilation, and hormone signalling, VOCs might reconfigure the primary and secondary metabolism to support physiological adjustments in receiver plants. Physiological adjustments to VOCs are characterized by an increase in defences before and upon stress in receivers, such as a greater production of extrafloral nectar (Kost and Heil, 2006; Choh *et al.*, 2006), volatile emissions (Engelberth *et al.*, 2004; Li and Blande, 2017), and proteinase inhibitors (Farmer and Ryan, 1990; Kessler *et al.*, 2006). VOCs can also influence receiver plant performance by affecting root and shoot growth (Ninkovic, 2003; Engelberth and Engelberth, 2019) and their reproduction (Kost and Heil, 2006; Pashalidou *et al.*, 2020).

A later component of the plant response to VOC cues is the regulation of the phytohormone network that leads to the induction or priming of plant defences, especially by promoting JA signalling (Arimura *et al.*, 2000*b*; Rodriguez-Saona *et al.*, 2009; Frank *et al.*, 2021). JA signalling plays a key role in inducing defensive responses locally and systemically upon insect feeding (Farmer *et al.*, 1992). Many GLVs have been shown to promote JA levels in receiver plants, which prepare defences for subsequent herbivore attack (Engelberth *et al.*, 2004; Frost *et al.*, 2008). (*Z*)-3-hexenol and (*Z*)-3-hexenyl acetate enhanced JA production in *Zea mays* after short-term exposure periods (Engelberth *et al.*, 2004). Several terpenes were also found to promote the JA pathways. The first observation was made in the year 2000 in a study in which several terpenes induced up-regulation of the lipoxygenase (*LOX*) gene as well as multiple defence genes mediated by JA in receiver plants (Arimura *et al.*, 2000*b*). More recently, (*E*)-nerolidol was found to increase JA and salicylic acid (SA) levels in *C. sinensis* (Chen *et al.*, 2020). SA signalling was also induced by exposure to volatilized α-pinene and β-pinene, which activated the expression of SAR-associated genes (Riedlmeier *et al.*, 2017). To date, several studies have shown that VOCs modulate JA, SA, and auxin phytohormonal pathways to enhance plant defences (Erb, 2018). Phytohormones are key elements of the signal transduction leading to the control of gene expression and the production of primary and secondary metabolites (Zhao *et al.*, 2005). Consequently, VOCs may act as modulators of receiver plant homeostasis and could trigger reconfiguration of receiver plant primary and secondary metabolism, thus enabling fine-tuning of responses in accordance with the situation represented by the VOC blend (Fig. 1).

Further events in the defence response include the enhancement of defensive gene expression, and an increase in secondary metabolites, including VOC emissions (Table 1). Several studies have reported the up-regulation of defence-related genes (Table 1), indicating that plants have detected and responded to a volatile cue, but falling short of demonstrating a tangible defence-related response. Moreover, gene expression may be more likely to be observed if analyses are conducted on excised leaves rather than whole plants, which was the case in several of the early studies (Baldwin *et al.*, 2002), although it should be noted that many more recent studies utilizing whole plants or seedlings have also shown the up-regulation of defence-related genes in response to volatile cues (Farag *et al.*, 2005; Godard *et al.*, 2008). For example, cabbage (*B. oleracea*) plants exposed to neighbours infested with *Pieris brassicae* larvae had higher levels of *LOX* transcripts than control plants (Peng *et al.*, 2011). After subsequent infestation with larvae, those same plants were more attractive to *Cotesia glomerata* parasitoids than controls, showing that an increase in gene expression can indicate a defence-related response. Increases in secondary metabolites such as proteinase inhibitors (Kessler *et al.* 2006) and Sporamin protease inhibitor (Meents *et al.*, 2019) were also reported. Several studies also showed that exposure to VOCs induced the *de novo* production of VOCs (Engelberth *et al.*, 2004; Yan and Wang, 2006; Cascone *et al.*, 2015) and increased secretion of extrafloral nectar as a source of sugar to attract more predators of herbivores (Kost and Heil, 2006; Heil and Silva Bueno, 2007; Li *et al.*, 2012). In a study of hybrid aspen (*Populus tremula* × *tremuloides*), the secretion of extrafloral nectar was directly induced by VOCs from herbivore-infested conspecifics, whereas increased emission of volatiles was primed and increased only upon subsequent herbivore feeding (Li *et al.*, 2012).

### Priming: responses to VOCs upon stress

Defence priming in plants was reported by Engelberth *et al.* (2004), whereby corn seedlings exposed to GLVs emitted by damaged neighbours increased their production of JA and volatile sesquiterpenes upon exposure to mechanical damage and regurgitant of *Spodoptera exigua*, the beet armyworm. This was a landmark study showing that plants can respond to an environmental cue to prepare themselves for defence without costly investment into manufacturing defence-related compounds in the absence of attack (Frost *et al.*, 2008). It was quickly suggested that utilizing herbivore-induced plant volatiles to prime agricultural plants for augmented defence expression could be an ecologically and environmentally sustainable method of combating pests (Turlings and Ton, 2006). Indeed, since the study by Engelberth *et al.* (2004) there has been a proliferation of work on defence priming (Table 1). Exposure to stress-induced volatiles has been shown to lead to quicker and stronger defence responses upon subsequent herbivory (e.g. Frost *et al.*, 2008; Cascone *et al.*, 2015; Shiojiri *et al.*, 2015) and increased resistance to herbivores, abiotic stresses (e.g. Cofer *et al.*, 2018), and pathogen infection (e.g. Girón-Calva *et al.*, 2012; Ameye *et al.*, 2015). Several studies have also shown that exposure to stress-induced volatiles makes receiver plants more attractive to beneficial insects or enhances the production of extrafloral nectar (Muroi *et al.*, 2011; Choh *et al.*, 2006). Focusing specifically on coverage of volatile-mediated priming, several reviews have provided detailed syntheses of current knowledge and insight into mechanisms and consequences (Frost *et al.*, 2008; Kant *et al.* 2009; Kim and Felton, 2013; Gonzalez-Bosch, 2018). However, there has been a rapid increase in reports of priming in the past few years, and knowledge on the intricacies of primed plant responses is increasing. Studies of maize have shown aboveground volatile emission and receipt of cues to be more important in volatile-mediated plant–plant interactions than belowground processes (van Doan *et al.*, 2021), while indole has been shown to be an important cue in priming of maize (Erb *et al.*, 2015) and tea (Ye *et al.*, 2021). Interestingly, priming in maize is induced independently by indole and the GLV (*Z*)-3-hexenyl acetate, but there is stronger priming when both molecules are present, in line with a synergistic effect (Hu *et al.*, 2019). A study by Michereff *et al.* (2021) indicated that priming in maize also appears to be genotype specific, although this observation was likely to be due to differences in the capacities of the two genotypes tested for induced volatile emission.

Interestingly, a recent study showed that whitefly infestation of tomato plants induces the release of a blend of volatiles that renders neighbouring plants more susceptible to whitefly infestation (Zhang *et al.*, 2019). The whitefly-induced volatiles prime SA-dependent defences and suppress JA-dependent defences, which renders the plants more suitable for whitefly development. Volatiles induced by the chewing herbivore *S. exigua* primed the plants for better defence against *S. exigua* larvae, indicated by lower weight gain of larvae. This suggests that the enhanced susceptibility induced by whitefly-induced volatiles is not a general response but could be part of an elaborate manipulation of the plants through low-molecular-weight proteins in the whitefly saliva (Xu *et al.*, 2019).

There is substantially less documented evidence of belowground volatile-mediated plant–plant interactions, although there is significant evidence indicating roles for root exudates and mycorrhizae in providing between-plant cues and signals (e.g. Babikova *et al.*, 2013). Recent studies have shown that sesquiterpenes constitutively released from roots of spotted knapweed, *Centaurea stoebe*, have no significant effect on the secondary metabolites of *Taraxacum officinale* plants (Huang *et al.*, 2019). A similar observation was observed in maize roots, whereby volatiles induced by the root-feeding banded cucumber beetle did not induce responses in neighbouring conspecifics (van Doan *et al.*, 2021). Studies of plant–plant interactions, and particularly priming, are potentially sensitive to the timing of the cue or signal, the longevity of the receiver plant response, and the plant organ receiving the cue. Consequently, to accurately detect priming there is a need to acquire temporal and spatial measurements of metabolic change. Non-invasive methods to detect physiological plant responses are required. Observing changes in VOC emission patterns over time can be done using proton transfer reaction mass spectrometry, which offers a means to monitor rapid changes in volatile emissions (Misztal *et al.*, 2016). Moreover, there is some potential for hyperspectral imaging to be utilized to correlate leaf reflectance to phytochemical data (Ribeiro *et al.*, 2018), which could be a useful tool for comparing plants exposed to different treatments, including volatile exposure and herbivore feeding. Non-destructive tools could be highly advantageous for measuring primed plant responses.

## Effect of VOCs on plant performance

### Effects on growth and reproduction

Typically, research on plant–plant interactions based on VOCs has focused on defence-related responses, which is a logical approach if it is considered that the VOCs emitted by herbivore-damaged plants are indicative of a potential attack (Douma *et al.*, 2019). The most appropriate response in a receiver plant could be to initiate or prepare defences to repel or minimize damage (Frost *et al.*, 2008). In this context, growth and reproduction have received less attention (Table 2). Growth responses could indicate possible competition-related or strengthening responses, whereas advanced flowering could indicate the preparation of damage-limitation strategies based on ensuring reproduction (Lucas-Barbosa *et al.* 2013).

**Table 2.** Summary of studies published on the effects of exposure to VOCs on plant primary metabolism and performance

Volatiles	Species	Exposure time	Effects on plant primary metabolism and performance	References
(*Z*)-3-Hexenyl acetate (*E*)-3-Hexenyl acetate	*Zea mays*	1.5 h	Increased growth	Cofer *et al.*, 2018
	*Z. mays*	16 h	Enhanced overall growth	Engelberth and Engelberth, 2019
	*Phaseolus lunatus*	7 d	Increased growth and flower production	Freundlich *et al.*, 2021
	*Capsicum annuum*	7 d	Reduced growth	Freundlich *et al.*, 2021
Clipped *Artemisia tridentata*	*A. tridentata*	24 h	Reduced growth in height	Karban, 2017
Clipped *A. tridentata*	*Nicotiana attenuata*	Growing season	Increased seed production	Karban and Maron, 2002
VOCs from *Solidago altissima* primed by *Eurosta solidaginis* volatiles	*S. altissima*	24 h	Increased growth rate and reduced rhizome production, indicating a reduction in clonal reproductive capacity	Yip *et al.*, 2019
Synthetic VOC blend	*P. lunatus*	3 d	Increased number of leaves and inflorescences	Kost and Heil, 2006
*Pieris brassicae* oviposition-induced volatile cues	*Brassica nigra*	5 d	Reduced growth and increased number of flowers and seeds	Pashalidou *et al.*, 2020
Salt-induced VOCs	*Vicia faba*	2 weeks	Reduced net photosynthesis; increased growth and resilience upon salt stress	Caparrotta *et al.*, 2018
VOCs from *Hordeum vulgare* cv. Alva	*H. vulgare* var. Kara	Throughout the vegetative growth phase	Increased root biomass and leaf area	Ninkovic, 2003
VOCs from another cultivar	*H. vulgare*	5 d	Reduced leaf temperature	Pettersson *et al.*, 1999
Jasmonic acid (25mM)	*Brassica napus*	Exogenous applications	Increased net photosynthesis, stomatal conductance, transpiration rate, and intracellular CO_2_ concentration	Ali *et al.*, 2018
Methyl salicylate (10mM)	*Vaccinium myrtillus*	Exogenous applications	Reduced net photosynthesis and down-regulated genes associated with growth, photosynthesis, and reproduction	Benevenuto *et al.*, 2019
Root VOCs of *Centaurea stoebe*	Several sympatric neighbours	Throughout growth	Increased germination and growth of sympatric neighbours	Gfeller *et al.*, 2019
Root VOCs of *C. stoebe*	*Taraxacum officinale*	7 weeks	Increased root protein, fructose, and sucrose concentrations	Huang *et al.*, 2019

Growth and reproduction are the most important components of plant fitness and constitute key physiological parameters to estimate the allocation of carbon. Since priming is an inducible phenomenon, theories predict a cost to inducing physiological changes (Douma *et al.*, 2017). This cost, although less expensive than induced defence, could reduce the resources allocated to growth and reproduction and in so doing affect plant fitness (Paul-Victor *et al.*, 2010; Orrock *et al.*, 2018). We previously stated that VOCs modulate JA signalling involved in the induction of defences, and it is mostly assumed that by enhancing JA signalling and defences, VOCs would inhibit growth (Zhang and Turner, 2008).

The cost of priming has been studied by assessing plant growth after the application of a low-dose of (*Z*)-3-hexenyl acetate to lima bean (*Phaseolus lunatus*) and pepper (*Capsicum annuum*) plants (Freundlich *et al.*, 2021). Whereas (*Z*)-3-hexenyl acetate-treated pepper plants showed reduced growth but had no difference in resistance to herbivores relative to controls, (*Z*)-3-hexenyl acetate-treated lima bean plants grew more, produced more flowers, and suffered less chewing herbivory compared with control plants (Freundlich *et al.*, 2021). Similarly, Karban (2017) found differential effects of exposure to volatile cues on plant growth depending on the species exposed. Young focal sagebrush (*Artemisia tridentata* subsp. *vaseyana*) plants exposed to clipped conspecific neighbours grew less in height than controls not exposed to these cues, whereas *Nicotiana attenuata* exposed to clipped sagebrush produced more seeds relative to control plants (Karban and Maron, 2002). These studies support the hypothesis that exposure to plant volatiles affects plant fitness (i.e. growth and reproduction) but that the extent to which fitness is affected differs depending on the species and the volatile chemical. This idea is corroborated in tall goldenrod with priming cues derived from *Eurosta solidaginis*; in a semi-natural field experiment, primed plants were shown to grow faster than unprimed plants but they produced fewer rhizomes, indicating a reduction in clonal reproductive capacity (Yip *et al.*, 2019).

Additional studies have provided evidence that plant volatiles might affect plant performance. Lima bean shoots exposed to HIPVs produced more leaves and inflorescences than untreated control shoots (Kost and Heil, 2006). More recently, it was reported that (*Z*)-3-hexenyl acetate-treated maize seedlings had a significant reduction in growth over the first 16h of treatment (Engelberth and Engelberth, 2019). However, the growth rate of seedlings increased on the second day after treatment, and their resistance against subsequent herbivory was not affected. At the end of the experiment, treated plants had similar or even slightly enhanced overall growth compared with control plants. A recent study on brassicaceous plants showed that oviposition-induced volatile cues ramp up plant defences; this was evaluated through performance assays with larvae of the specialist *Brassica*-feeding herbivore *P. brassicae* (Pashalidou *et al.*, 2020). There was a concurrent reduction in the growth of receiver plants, but an increase in the number of flowers and seeds (Pashalidou *et al.*, 2020).

### Effects on gas exchange and nutrient uptake

The literature suggests that plants may have developed several physiological mechanisms to compensate for the cost of inducing defences (Engelberth and Engelberth, 2019). Plants might increase their photosynthesis or stomatal conductance (to take up more CO_2_) or increase their root length (to take up more nutrients). Interestingly, salt stress has been shown to induce volatile emissions that can prime neighbouring plants for greater resilience to salt in Arabidopsis (Lee and Seo, 2014), broad bean (*Vicia faba*) (Caparrotta *et al.*, 2018), and sweet basil (*Ocimum basilicum*) (Landi *et al.*, 2020) plants. Broad bean plants showed reduced photosynthesis in response to VOCs but gained greater resilience to salt and grew more than controls upon salt exposure (Caparrotta *et al.*, 2018). Sweet basil did not reduce photosynthesis upon receipt of a volatile cue, but when subsequently exposed to salt it reduced photosynthesis more dramatically than controls, maintained a greater water use efficiency, and, while not differing from controls in growth, showed earlier senescence and flowering, and a greater seed set (Landi *et al.*, 2020).

Ninkovic (2003) investigated the effect of volatiles from two barley (*Hordeum vulgare*) cultivars (Alva and Kara) on plant root and shoot biomass, and found that Kara plants exposed to Alva volatiles allocated significantly more biomass to roots and increased their leaf area compared with Kara plants exposed to volatiles from Kara or to clean air. Another study on different barley cultivars reported a reduction in leaf temperature when some cultivars were exposed to volatiles from other cultivars (Pettersson *et al.*, 1999). A reduction in leaf temperature is generally correlated with a higher transpiration rate and greater stomatal conductance. A greater stomatal conductance assumes an increase in CO_2_ uptake, which is required for a higher photosynthetic activity. Recent work investigating the effect of HIPVs on the photosynthesis and stomatal conductance of receiver plants has indicated increases of both relative to controls (Blande and Yu, 2021). With the exception of this ongoing research, the effect of plant volatiles on photosynthesis has been observed in connection with exogenous application of the signalling phytohormones SA and its volatile derivative methyl salicylate (MeSA), and JA and its derivative MeJA. The exogenous application of JA (25mM) was found to increase net photosynthesis, stomatal conductance, transpiration rate, and intracellular CO_2_ concentration (Ali *et al.*, 2018). However, the application of MeSA (10mM) and MeJA (150mM) decreased photosynthesis and down-regulated genes associated with growth, photosynthesis, and reproduction (Rahnamaie-Tajadod *et al.*, 2017; Benevenuto *et al.*, 2019).

## Ecological implications of responding to VOCs

**Fig. 2.**
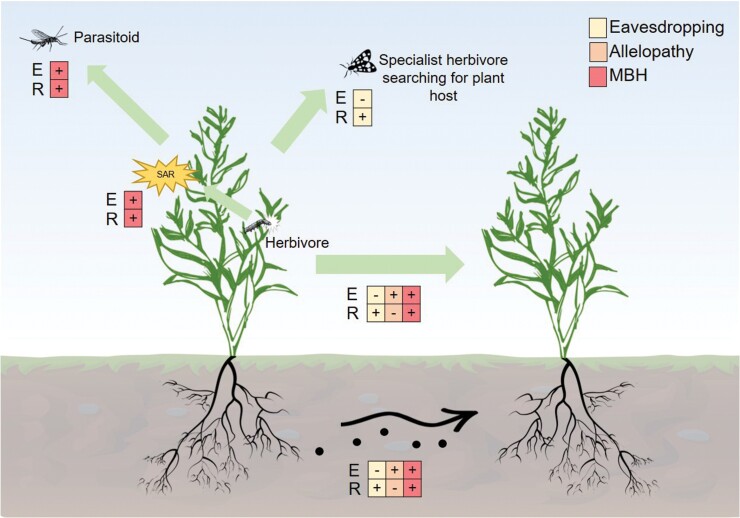
Ecological consequences of emitting and receiving volatile cues. The arrows indicate the direction of VOC transport. The boxes indicate the potential outcomes of the response and whether the emitter or receiver gains a beneficial (+) or a detrimental (-) effect on fitness. SAR indicates that the emitting plant may gain systemic acquired resistance. We refer to allelopathy as a phenomenon whereby the emitter releases chemicals that have detrimental effects on the performance of the receiver plant (Inderjit and Duke, 2003). Eavesdropping is the process whereby a receiver intercepts and uses information encoded in chemical cues that evolved to provide information to a different recipient (Karban, 2015). MBH indicates the mutual-benefits hypothesis, whereby the emitter and receiver benefit from the transport of VOC cues, irrespective of their relatedness, through the responses of receivers reducing the risk of herbivory (Kalske *et al.*, 2019).

In the above sections we have provided an overview of mechanisms underpinning volatile-mediated plant–plant interactions and the variety of responses induced by VOCs in relation to both plant defences and performance. It is evident that a number of responses induced in receiver plants can affect the behaviour and performance of herbivorous insects (e.g. Grof-Tisza *et al.*, 2020) and plant resistance to other biotic or abiotic stressors (e.g. Cofer *et al.*, 2018). Therefore, the role of volatile-mediated plant–plant interactions can be of ecological importance (Fig. 2). A number of studies conducted under field conditions have shown that plants receiving damage-induced volatile cues can gain a greater level of resistance to pests during the course of a season (e.g. Tscharntke *et al.*, 2001; Karban *et al.*, 2006). It has been argued that receiver plants ‘eavesdrop’ on cues that have evolved for different recipients, for example, predatory or parasitic insects, or different parts of the emitting plant (Fig. 2). However, a number of nuances in plant–plant interactions have been observed indicating that there could be a higher than expected level of complexity and sophistication. Sagebrush plants have been shown to produce cues that are responded to more strongly by self-cloned plants than by non-self alternatives (Karban and Shiojiri, 2009). Sagebrush chemotypes can also respond more effectively to volatiles emitted by individuals of the same, rather than a different, chemotype (Karban *et al.*, 2014*a*). Moreover, it seems that some chemotypes may be better at receiving cues than others. Maize genotypes that differ in traits related to volatile emission capacity also appear to differ in their abilities to interact (Michereff *et al.*, 2021). Interspecific interactions have also indicated that some plants are better senders and receivers of cues than others (Karban *et al.*, 2006; Pearse *et al.*, 2012). It appears that although the quantity of volatiles emitted could play a significant role in determining what makes a good emitter, it does not follow that all plants receiving the cues from such an emitter will respond.

A recent study by Kalske *et al.* (2019) tested the predictions of two hypotheses, the kin-selection and mutual-benefits hypotheses, to determine the selective criteria that would favour the occurrence of volatile-mediated plant–plant interactions. The kin-selection hypothesis dictates that emitter plants would indirectly benefit from volatile-mediated interactions by providing a cue that improves the fitness of their kin more than that of other genotypes. The mutual-benefits hypothesis requires that the emitter plant benefits from providing cues to plants irrespective of their relatedness, through the responses of receiver plants reducing the risk of herbivory (Fig. 2). Using tall goldenrod (*Solidago altissima*) as a model system, the communication of plants in an area with regular herbivore infestation was compared with that of those in an area from which herbivores were excluded. The authors observed that when selection occurred under the condition in which herbivores were excluded, interactions between related plants were more effective, whereas when selection occurred with herbivores present, communication was more uniform throughout the population (Kalske *et al.*, 2019). An earlier study showed that plant–plant interactions in tall goldenrod led to herbivores spending less time on a receiver plant and causing less damage (Morrell and Kessler, 2017). These observations provide some of the most compelling evidence for plant–plant interactions to be considered a true communication process. While there have been a number of field studies investigating volatile-mediated plant–plant interactions, relatively few have extended to the point of examining the fitness costs and benefits. There is a clear need for a better integration of responses based on growth or reproduction with the modulation of direct and indirect defences.

Assessing the fitness benefits of plant–plant interactions provides a technical challenge due to the difficulties in manipulating plants that can and cannot interact via volatiles, while maintaining ecologically realistic scenarios. Advances in genomics in the past couple of decades have enabled the development of ‘deaf’ or ‘mute’ plants that cannot detect volatile cues (deaf) or do not release detectable cues (mute) (Baldwin *et al.*, 2006). It was proposed that these advancements would enable the research community to test whether volatile-mediated interactions between plants enhance the fitness of plants in natural communities (Baldwin *et al.*, 2006; Dicke and Baldwin, 2010). A study conducted with *N. attenuata* in nature showed that mute plants, those silenced in HIPV emission, have lower fitness than HIPV-emitting plants, based on the recruitment of *Geocoris* spp. predators (Schuman *et al.*, 2012). However, field studies utilizing such mute or deaf plants to develop understanding of the ecological implications of volatile-mediated plant–plant interactions remain mostly lacking. Indeed, mechanistic studies to explicitly determine the roles of chemical mediators of plant interactions under field conditions in general are typically lacking (Schuman and Baldwin, 2018).

Since the chemotype of plants appears to be an important factor in determining the outcome of intraspecific plant–plant interactions, there is scope for increasing our understanding of the intricacies of plant–plant interactions through reciprocal experiments exposing numerous different chemotype receivers to numerous different chemotype emitters. Combining the use of chemotypes with the genomic tools for creating mute and deaf plants would provide an excellent resource for future studies on the ecology and mechanisms of variation in intraspecific plant–plant interactions.

## Conclusions and future directions

Here, we have shown that the perception of VOCs by plants triggers a series of events leading to changes in plant defence and performance. The induction or priming of defences depends on the intake or storage of energy, and the availability of carbon, nitrogen, and sulfur provided by primary metabolism. In this review, we highlighted that VOCs have the potential to affect the photosynthesis rate and nutrient uptake and therefore modulate primary and secondary metabolisms, leading to change in overall plant performance (Fig. 1).

Evidence is accumulating that volatile-mediated interactions are nuanced and highly variable based on the capacity of plants to produce and emit volatiles, but there are as yet several poorly elucidated factors. The trade-off between growth and defence is important, particularly given that rapid growth is a desirable trait in many agricultural crop species that have been models in studies of plant–plant interactions. Our literature searches indicated that most studies that have looked at the growth of plants after their exposure to plant VOCs concern the phenomenon of allelopathy (Fig. 2). Relatively few studies have focused on this goal in the context of other plant–plant interactions. This indicates that an excessively strong focus on defence-related pathways could miss important responses in primary metabolism, growth, and reproduction. Measurements of plant reproductive capacity or success are critical for determining the potential fitness benefits of plant–plant interactions. There are some non-invasive techniques for monitoring photosynthesis and stomatal conductance, which can indicate changes in the primary metabolism of plants. Future research should in general capitalize on technological advancements for the non-destructive measurement of plant responses, which will enable a deeper understanding of temporal and spatial receiver plant responses. It has been shown that volatile-mediated plant–plant interactions occur both above and below ground, but there is significant variation in the efficiency of each for different species, and even for different genotypes of the same species. Hence, finally, we propose that future research focuses more on integrating concurrent above- and belowground processes and the trade-offs in each occurring under different environmental conditions.
